# Surface-confined FRET nanoplatform printed *via* pyro-EHD jet for stable and reproducible TNF-α detection

**DOI:** 10.1039/d6ra00144k

**Published:** 2026-04-20

**Authors:** Stefania Carbone, Simone Russo, Anna Palma, Giuseppe Junior Mosca, Sara La Manna, Alessia Cugudda, Sara Coppola, Pier Luca Maffettone, Simonetta Grilli, Giuseppe Vitiello, Concetta Di Natale

**Affiliations:** a Department of Chemical, Materials and Industrial Production Engineering, University of Naples Federico II Piazzale Tecchio 80 80125 Naples Italy giuseppe.vitiello@unina.it concetta.dinatale@unina.it; b Institute of Applied Sciences and Intelligent Systems (ISASI), National Research Council of Italy (CNR) Pozzuoli NA 80078 Italy simonetta.grilli@cnr.it; c Center for Colloid and Surface Science (CSGI) via della Lastruccia, Sesto Fiorentino FI 80078 Italy; d Department of Pharmacy, University of Naples Federico II 80131 Naples Italy

## Abstract

The detection of protein biomarkers at low concentrations through fluorescence resonance energy transfer (FRET) remains challenging, as conventional homogeneous assays are often affected by uncontrolled donor–acceptor diffusion, fluorophore quenching, and limited reproducibility. In this work, we propose a two-dimensional solid-supported FRET nanoplatform enabling a surface-confined sensing strategy for the stable and reliable detection of the model biomarker Tumor Necrosis Factor-alpha (TNF-α). The approach combines a nanostructured film of fluorine-doped ZnO (F/ZnO) quantum dots deposited on a glass slide with a high-precision pyro-electrohydrodynamic jet (p-jet) printing technique. Microspots of a high-affinity peptide (P52) were printed onto the F/ZnO layer to control donor–acceptor spacing and optimize fluorophore orientation and density, ensuring efficient FRET and reduced variability between samples. The platform provides stable, reproducible, and concentration-dependent FRET signals in the ng mL^−1^ range, with a limit of detection of 31 ng mL^−1^, suitable for identifying elevated cytokine levels associated with inflammatory responses. Assay selectivity was evaluated in the presence of non-target proteins, including bovine serum albumin (BSA) and phosphorylated Tau (p-Tau181), and in artificial urine as a complex biological matrix. The results indicate limited interference and minimal matrix effects. Overall, this strategy offers a robust architecture with low reagent consumption and scalable fabrication for future point-of-care diagnostic applications.

## Abstract 2

A p-jet/F-ZnO 2D FRET nanoplatform enables surface-confined TNF-α detection by printing P52 microspots with fixed donor–acceptor spacing. The system ensures high reproducibility and sensitivity, achieving a 31 ng mL^−1^ limit of detection.

## Graphical abstract

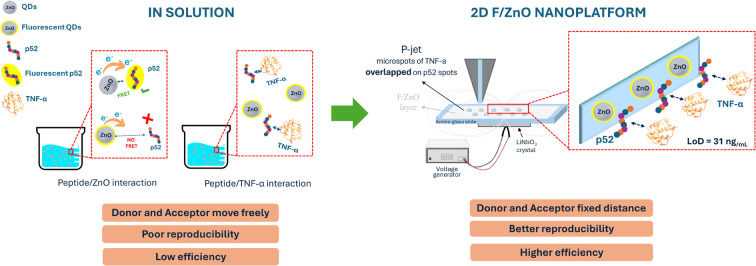

## Introduction

In recent years, the demand for highly sensitive and selective protein biosensing platforms has grown significantly, driven by emerging needs in biomedical research.^1,2^ However, the detection of proteins at low concentrations remains a significant challenge, requiring the development of novel sensing technologies that offer improved performance. Fluorescence Resonance Energy Transfer (FRET) is one such technique that has garnered considerable attention due to its ability to monitor molecular interactions at the nanometer scale.^3,4^ FRET-based sensors offer high sensitivity and rapid response times, as well as the potential for real-time analysis.^5,6^ Despite their advantages, conventional FRET assays performed in homogeneous solution systems present several drawbacks that limit their practical utility in biosensing applications. In solution, donor and acceptor molecules undergo random diffusion, leading to uncontrolled intermolecular distances and orientations that remain critical for effective energy transfer. This results in low FRET efficiency and high variability in signal output.^6,7^ Moreover, aggregation and fluorophore quenching are usually intensified in free-solution environments, reducing the overall signal stability and lifespan of the sensor. The dynamic nature of the molecular interactions in solution also complicates the quantitative analysis, particularly at low analyte concentrations.^6,8^

Conversely, implementing FRET on nanostructured two-dimensional (2D) surfaces offers several distinct advantages, such as fixed spatial arrangement between donor and acceptor molecules, enhanced photostability due to surface passivation effects, and improved control over molecular orientation and density.^9–13^ The high surface-to-volume ratio of 2D nanostructures enables biomolecules to be immobilized in an organized manner, thereby increasing the probability of productive FRET interactions. Additionally, the effects of surface-enhanced energy transfer and the reduced level of background fluorescence further contribute to higher sensitivity and signal reproducibility.^14,15^ Such benefits make the 2D nanostructured platforms particularly suitable for the development of ultrasensitive surface-based FRET biosensors. ZnO has been extensively studied as a FRET-enhancing material due to its wide bandgap, strong fluorescence, photostability, biocompatibility, and easy surface functionalization. The advent of 2D ZnO nanostructures provided large active surfaces for biomolecule immobilization and improved probe interactions, enabling ultrasensitive and specific biosensing.^16^ Their direct bandgap (3.37 eV) supports intense, stable near-band-edge emission suitable for FRET.^17^ Bandgap tuning *via* size control or doping (*e.g.*, Mn^2+^, Co^2+^, Ni^2+^) suppressed surface traps and improved optical behaviour.^18^ The wurtzite structure imparts intrinsic piezoelectricity, useful for self-powered biosensors.^19^ Peptide functionalization exploited electrostatic/coordination interactions with ZnO, with residues like arginine enhancing stability and fluorescence readout.^20^ Uniform 2D films improved reproducibility and bioreceptor immobilization,^21^ and their fabrication *via* drop casting or spin coating enabled scalability.^22–24^ ZnO-based FRET biosensors were also integrated into portable/wearable devices for real-time, non-invasive biomarker monitoring in personalized medicine.

In the recent years, we introduced the pyro-electrohydrodynamic jet (p-jet) technique which can print with high spatial precision microspots of biomolecule solutions on solid supports, such as reactive glass slides. This technique exploits the pyroelectric effect of a LiNbO_3_ crystal for nozzle-free, contactless and electrode-free deposition of sub-pL droplets onto a sensor surface.^25^ Upon thermal stimulation, the crystal generates an electric field that interacts with the meniscus of a mother drop of sample, accumulating charges at the interface, where the resulting Coulomb repulsion induces the formation of the so-called Taylor cone. Such electric stress increases and, when overcoming the surface tension of the liquid, leads to tiny droplet ejection.^26,27^ Compared to conventional inkjet systems,^28^ the p-jet enables precise droplet overlapping, critical for accumulating low-abundance analytes in biosensing. This technique was successfully used to enhance biomarker concentration and achieve sub-picogram detection levels.^27,29,30^

In this work, we adopted the p-jet for developing an innovative 2D F/ZnO nanoplatform for highly sensitive FRET detection of a model biomarker represented by the Tumor Necrosis Factor-alpha (TNF-α), a pro-inflammatory cytokine linked to cancer progression and poor prognosis.^31^ We exploited the high spatial precision of p-jet to print microspots of a peptide with high affinity toward TNF-α on a glass slide pre-coated with a thin film of fluorine-doped ZnO (F/ZnO) quantum dots^32^ and, successively, a precisely overlapped microspot of the analyte (*i.e.* TNF-α). The high surface-to-volume ratio and isoelectric point of the F/ZnO film enabled stable peptide immobilization. Serial dilutions of TNF-α were deposited onto the spots, producing a net fluorescence response inversely proportional to the analyte concentration. Compared to solution-based FRET assays, the 2D F/ZnO nanoplatform proposed here minimizes the diffusion limits, the fluorophore aggregation, and the nanoparticle precipitation, enhancing the energy transfer efficiency. The fixed donor–acceptor arrangement confines the FRET signal, improves reproducibility, and reduces background noise.^9^ Our present work aims to demonstrate the stability, reproducibility, and feasibility of a surface-confined FRET architecture enabled by precision p-jet printing, rather than competing with established ELISA platforms in terms of sensitivity. We report ng mL^−1^-level detection of the TNF-α biomarker as a first demonstration of a solid-supported FRET approach, while acknowledging that conventional ELISA methods routinely achieve pg mL^−1^ sensitivity.^29^ To approximate a complex biological matrix, measurements in artificial human urine were performed to assess and assessed selectivity in the presence of representative protein interferents (*e.g.*, bovine serum albumin (BSA) and phosphorylated Tau (p-Tau181)). These tests were designed to evaluate the impact of potential off-target binding and matrix-driven effects on the FRET response. We believe that the innovative p-jet technique applied to FRET-based assays could open the route for early-stage disease diagnostics.

### Materials and methods

#### Materials

Zinc acetate dihydrate, Zn (CHCOO)_2_·2H_2_O (purity 99%), ammonium hydrogen difluoride NH_4_HF_2_ (purity 99.999%), sodium hydroxide KOH (purity 85%), methanol (purity 99.8%), and ethanol (96% vol.) were purchased from Merck (Germany) and were used without any further purification. Reagents for peptide synthesis were obtained from Iris Biotech (Germany). Solvents for peptide synthesis and HPLC analyses were from Romil (Dublin, Ireland). Reversed phase columns for peptide analysis and the LC-MS system were from ThermoFisher (Waltham, MA). The TNF-α (recombinant human TNF alpha protein (active)) was purchased from Abcam, UK (ab259410), and was reconstituted with 1 mL of Phosphate-Buffered Saline (PBS, from Merck/Sigma-Aldrich, Italy) 1× pH 7.4 to have a final concentration of 0.14 mg mL^−1^ for the mother solution. Biological matrix testing was performed using artificial human urine (Biochemazone, BZ325; Chemazone Inc., Alberta, Canada) as a standardized urine surrogate for sample analysis. Per the manufacturer's certificate of conformity, it consists of urea, creatinine, and major urinary ions (Cl^−^, Na^+^, K^+^, Ca^2+^, SO_4_^2−^, NH_4_^+^, PO_4_^3−^, Mg^2+^) in deionized water, with trace human serum albumin and 0.05% preservative. The formulation is designed to mimic urine from healthy individuals based on literature/official references. Specificity was assessed by testing the target analyte alongside potential interfering proteins, including bovine serum albumin (BSA) and phosphorylated Tau at threonine-181 (p-Tau181). BSA was purchased from Merck/Sigma-Aldrich (Italy), and p-Tau181 was obtained from Fujirebio (Japan). BSA and p-Tau181 were tested at final concentrations of 100 ng mL^−1^ and 2 pg mL^−1^, respectively, in artificial human urine (Biochemazone, BZ325) diluted 1 : 10 (v/v) in 1× PBS. Glass slides functionalized with 2D-Amine (PolyAn GmbH, Germany), with a standard size of 25 × 75 × 1 mm, were purchased from Arrayit Corporation. The SMP3 printing pin was purchased from ArrayIt Corporation, originally designed for contact printing on microarray slides. The tip of the pin had an external diameter of 80 µm and an internal diameter of 20 µm. The LN was purchased from Crystal Technology Inc. in the form of 500 µm c-cut 3-inch wafers that were then cut into pieces (2 × 2) cm^2^ sized by a standard diamond precision saw. At equilibrium, the spontaneous polarization Ps of a c-cut LN crystal is fully compensated by screening charges from the environment. According to the pyroelectric effect, a temperature change Δ*T* causes a variation Δ*P*_s_ ∝ Δ*T*. This builds up an uncompensated surface charge *σ* ∝ *p*_c_Δ*T*, where *p*_c_ is the pyroelectric coefficient,^33,34^ that generates a high electric field.^35^ The high-speed camera used for monitoring the spot formation was a Thorlabs Quantalux® Scientific CMOS (sCMOS) Camera, which boasts a resolution of 1920 × 1080 pixels (2.1 megapixels) and was equipped with a 10× microscope objective.

#### Synthesis of TNF-α selective peptide

The FITC-labelled peptide P52 (FITC-βA-HAIYPRH-NH_2_) was selected for its high affinity toward TNF-α, as demonstrated in previous phage display screenings that identified this sequence as containing key residues compatible with the binding motif to TNF-α.^36^ The FITC-labelled peptide P11 (FITC-βA-ITPAHMD-NH_2_), which lacks these critical residues, was used as a negative control to confirm binding specificity. P11 and P52 peptides were synthesized as C-terminal amides on Rink-amide resin (substitution 0.57 mmol g^−1^, 25 µmol scale), using the 9-fluorenylmethoxycarbonyl/*tert*-butyl (Fmoc/*t*Bu) strategy, as described before.^37^ They were then purified by preparative Reverse-Phase (RP)-High-Performance Liquid Chromatography (HPLC), applying a linear gradient of 0.1% trifluoroacetic acid (TFA) CH_3_CN in 0.1% TFA water from 5–70% over 20 min with a semipreparative 2.2 × 5 cm C18 column at a flow rate of 15 mL min^−1^, using a UV detector set at a wavelength of 210 nm. Peptides' purity and identity were confirmed by Liquid Chromatography (LC)-Mass Spectrometry (MS). Finally, they were lyophilized and stored at −20 °C until use. The mother sample was diluted in Milli-Q water (control) to obtain the following concentrations: 2 mg mL^−1^, 0.8 mg mL^−1^, 0.6 mg mL^−1^, 0.4 mg mL^−1^, and 0.2 mg mL^−1^.

#### Preparation of F/ZnO nanostructured platforms by spin-coating F/ZnO

Fluorine-doped ZnO quantum dots (QDs) were synthesized through the wet-precipitation method utilized in previous works^38,39^ in order to show improved emission properties^38^ useful to design nanostructured devices for optical sensing QDs. For brevity we call them in this paper indifferently as QDs or simply F/ZnO. In summary, 1.842 g of zinc acetate precursor were dissolved into 74 mL of methanol within a three-neck round-bottom flask at 60 °C. A suitable quantity of fluorine doping agent, such as NH_4_HF_2_ salt, was subsequently added to the mixture to atomically doping ZnO QDs and improve the emission behaviour.^38^ As previously demonstrated, fluorine doping involves replacing some oxygen ions in the ZnO lattice with fluoride ions, thus introducing extra free electrons and regulating non-radiative defects which contribute to enhance the radiative recombination.^40,41^ Finally, 42 mL of 4 M KOH in methanol was added dropwise to the solution. The finished mixture was stirred at 60 °C for two hours and subsequently centrifuged three times at 10.000 rpm for five minutes, followed by resuspension in methanol. Two aliquots of 300 µL of F/ZnO suspension at two concentrations of 2 and 4.6 mg mL^−1^ were retrieved. According to a previously described approach^38,39^ these aliquots were spin-coated at 2.000 rpm for 150 s (spin coater WS-400-6NPP, Laurell Technologies Corporation) on two types of glass slides with a standard size of 25 × 75 × 1 mm to achieve nanostructured thin films: (1) standard microscope slide; (2) 2D-amine (see the previous section).

#### Peptide functionalization of the 2D-FRET nanoplatform by the p-jet technique

The F/ZnO film on the glass slides was functionalized with microscopic spots of the two labelled peptides P52 and P11 by the p-jet technique, using the setup already introduced and well described in ref. 30 and 42. Here we summarize the main features of the procedure. A volume of peptide solution of about 1 µL was loaded in the printing pin. Under the pin, we have the LN crystal and, underneath, a wire of tungsten (300 µm thick) bent to get a nearly point-wise heat source. Wire and crystal were aligned with the pin, and the glass slide was mounted on a three-axes translation stage to stand in between and to be translated for printing different spots. The wire was heated by the Joule effect through the flowing current induced by a voltage signal with amplitude 1.7 V, period 60 s and duty cycle 5%. This setting provided a temperature variation of about 10 °C from 25 to 35 °C at maximum. This variation was sufficient to establish the pyroelectric effect but avoiding potential denaturation for the biomolecules. The formation of the spots was monitored in real time using a compact scientific digital camera. The electric field generated by the crystal (see Material and methods section) makes charges accumulate on the meniscus of the liquid in the pin. The repulsion force deforms the liquid surface progressively to a conical tip, the so-called Taylor cone.^43,44^ The cone breaks when the electrical force is high enough to overcome the liquid surface tension, and an unstable liquid bridge forms and leaves a tiny droplet on the slide surface, forming the peptide microspot. After each spot, the pin was cleaned in an ultrasonic bath with Milli-Q water for a few minutes to minimize cross-contaminations. After printing, the slide was inserted into the scanner for recording the fluorescence images that were analysed by ImageJ, an open-source Java-based image processing program developed by the National Institutes of Health (NIH).

#### Analytical characterisation methods

Dynamic Light Scattering (DLS) measurements were carried out to investigate the assessment of the size distribution and stability in solution of the prepared F/ZnO QDs by utilizing the Zetasizer Ultra (Malvern Panalytical Ltd, Worcestershire, United Kingdom). UV-Vis's absorption and fluorescence emission spectra of suspensions containing bare F/ZnO QDs and in the presence of peptides P52 and P11 were recorded by using the FS6 spectrofluorometer (Edinburgh Instruments, UK). Scanning Electron Microscope (SEM) images were recorded to investigate the morphology of the nanostructured F/ZnO films on both bare and amino glass slides. The images were acquired using a Hitachi TM3000 Tabletop SEM under standard operating conditions. After deposition and drying, the coated slides were cut into approximately 1 cm^2^ sections and mounted on aluminium stubs for analysis. The Energy Dispersive X-ray (EDX) was also used to monitor the chemical composition of the obtained films. For chemical orientation and to study the effective nanoparticles and peptide interactions, FTIR-ATR measurements were performed using a Nicolet iS50 spectrometer to investigate the interaction between F/ZnO QDs and the peptide. Spectra were collected at a resolution of 4 cm^−1^ with 64 scans accumulated for each sample.

#### Fluorescence resonance energy transfer (FRET) assay for TNF-α detection

The interaction between the F/ZnO nanostructured films and the two peptides P52 and P11 was investigated first in a conventional solution environment and then in case of the innovative 2D nanoplatform proposed here. First, we prepared a solution of F/ZnO at a concentration of 0.05 mg mL^−1^ in methanol 98% and two aliquots of 2 mL were retrieved. Two different molar ratios of F/ZnO : peptide were tested for both peptides P52 and P11: (1) 1 : 0.1 ratio; (2) 1 : 1 ratio. In the first case we prepared the peptide solution at a concentration of 0.005 mg mL^−1^ in Milli-Q water, and we added aliquots of 2.5 µL to the F/ZnO solution to increase progressively the number of peptide equivalents from 0 to 1. In the second case the procedure was the same but by using a concentration of the peptide at 0.05 mg mL^−1^ in Milli-Q water. We recorded the emission spectra at the typical excitation wavelength of F/ZnO, namely 350 nm.^38^ In case of the innovative 2D nanoplatform proposed here, the images of the microspots were captured using a commercial confocal fluorescence scanner (Innoscan710, Innopsys). The 532 nm laser source was employed to excite the molecules in the spots, while the two digital photomultiplier tubes (PMT) in the scanner were utilized to detect the emitted fluorescence signals. The scanning parameters were set as follows: laser power at 5 mW, detection gain 1 (to avoid saturated pixels), scanning speed at 32 lines per s, and spatial resolution of 5 µm per pixel. The 16-bit TIFF images provided by the scanner were quantitatively analyzed using ImageJ software to determine the mean fluorescence signal intensity of each spot. The fluorescence intensity was retrieved as the average value over 20 replicated spots for each peptide and protein concentration.

#### Surface-confined FRET assay for TNF-α detection in buffer and artificial urine matrix

The FRET-based assay was carried out on amine-functionalized glass slides coated with a nanostructured ZnO thin film (F/ZnO platform), using the fluorescently labeled peptide P52 as the FRET donor. The P52 peptide was printed at 2 mg mL^−1^ onto the F/ZnO-coated slides using a p-jet microspotting system to create multiple replicated spots, followed by incubation for 24 h at 25 °C to ensure immobilization of the peptide onto the surface. After this step, serial dilutions of TNF-α (1–140 ng mL^−1^ in PBS) were then overprinted directly onto the P52 spots (10 replicates per concentration) using the same p-jet setup. Slides were incubated overnight to allow binding between TNF-α and the surface-confined F/ZnO–P52 complex. Following incubation, slides were rinsed with Milli-Q water to remove unbound material, dried under a gentle nitrogen stream, and re-imaged under the same scanner settings. The entire procedure was then repeated using biochemazone BZ325 artificial human urine, diluted 1 : 10 in PBS and supplemented with 100 ng per mL bovine serum albumin (BSA) and 2 pg per mL phosphorylated Tau (p-Tau181) to simulate biologically relevant interference. TNF-α dilutions were prepared in this complex matrix and subjected to the same overprinting, incubation, rinsing, imaging, and analysis workflow. Applying identical acquisition and quantification protocols in both standard and interferent-containing matrices enabled direct assessment of assay specificity, linearity, and robustness under realistic biological conditions. The background-corrected fluorescence data were used to construct calibration curves and to determine the limit of detection (LoD) and limit of quantification (LoQ), as detailed in the Results section.

#### Limit of detection (LoD) and limit of quantification (LoQ) evaluation

The mean fluorescence intensity of the spots was plotted as a function of the analyte concentration in the working range of 0 to 140 ng mL^−1^. The Limit of Detection (LoD) and Limit of Quantification (LoQ) of the assay were evaluated according to the ICH Guidelines^45^ as previously reported.^30,46^

## Results and discussion

### Design and optical characterization of peptide-based sensing nanoplatform

**Fig. 1:**
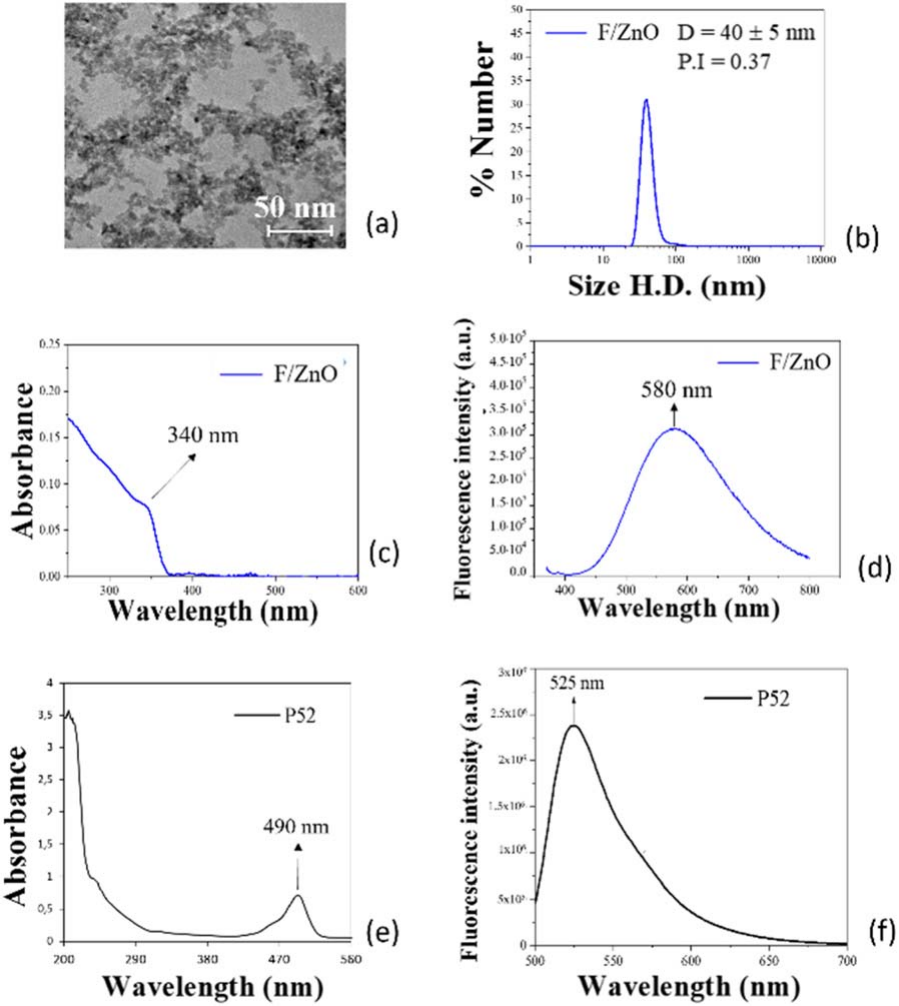
(a) F/ZnO QDs TEM images; (b) results of the DLS measurements on F/ZnO in solution with the behaviour of the autocorrelation function and of the hydrodynamic diameters respectively; (c) absorption spectrum of F/ZnO QDs; (d) fluorescence emission spectrum of F/ZnO QDs; (e) absorption spectrum of P52; (f) fluorescence emission spectrum of P52 excited at 490 nm.

A preliminarily spectroscopic characterization was performed for F/ZnO and for the peptide P52 in a solution environment, to evaluate the best conditions for achieving the FRET effect. The graphs in Fig. 1 show the resulting measurements, where Fig. 1(a–f) refer to the F/ZnO QDs and to the peptide P52, respectively. Fig. 1(a) shows a TEM image indicating the formation of QDs with a pseudo-spherical morphology and a mean size of about 5 nm in radius, in agreement with a previous study.^38^Fig. 1(b) presents the size distribution curve of F/ZnO QDs as obtained by DLS analysis which exert a monomodal character indicating the presence of a single population of F/ZnO QDs with hydrodynamic diameter of about 40–50 nm. This can be ascribable to the presence of small clusters formed in solution, due to the uncoated nature of QDs surface which favours the self-aggregation. By a optical point of view, the UV-Vis's measurement in Fig. 1(c) shows an absorption peak at 340 nm when excited at 490 nm, while typical values are reported in the range 360–370 nm.^47,48^ This absorption at a shorter wavelength is due to the quantum confinement effect, typical of QDs.^49,50^Fig. 1(d) and S1 reveal the fluorescence behaviour of the F/ZnO QDs excited at 350 nm, with a broad emission centered at 580 nm. Fig. 1(e and f) shows the UV-Vis absorption and fluorescence spectra of the P52 peptide, which exhibits an absorption band around 490 nm and an emission peak at 525 nm (upon excitation at 490 nm). These findings confirm a partial spectral overlap between the emission of the F/ZnO QDs and the absorption of P52, thus supporting the feasibility of a FRET interaction in the p-jet microspots. In this system, the F/ZnO QDs act as the FRET donor, while P52 serves as the acceptor. Although the spectral overlap is not optimal, it is sufficient to allow energy transfer under favourable conditions. The emission of F/ZnO QDs partially overlaps with the lower-wavelength region of the peptide absorption, enabling potential FRET interactions and indicating a close spatial proximity between the two components. However, as indicated by DLS data, Fig. 1b and S2, F/ZnO QDs tend to aggregate, which can partially affect FRET efficiency due to an uncontrolled donor–acceptor distance. Therefore, a 2D arrangement is preferred, as it offers better control over the spatial arrangement, enhancing the likelihood of effective energy transfer, creating more favourable conditions for FRET to occur. The 2D assay environment was then investigated by using glass slides coated with a thin film of F/ZnO QDs obtained by standard spin coating. As explained in the section Materials and methods, we used two kinds of commercially available slides, the standard microscope slide and the 2D-amine slide, for brevity amino slides. We tested two concentrations of F/ZnO at 2 mg mL^−1^ and at 4.6 mg mL^−1^ and we compared the stability and the homogeneity of the resulting coatings through the measurement of the contact angle of a sessile drop of Milli-Q water and through SEM/EDX acquisitions. Moreover, we recorded the fluorescence images of the slides by the scanner.

**Fig. 2:**
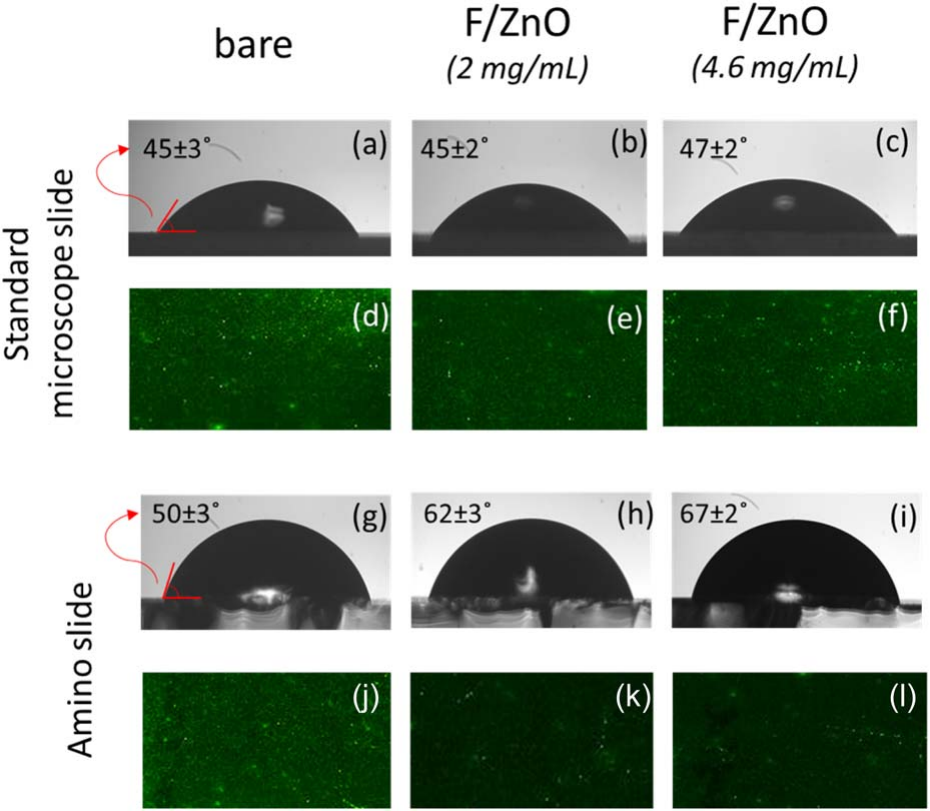
Typical images recorded on the glass slides for evaluating the contact angle and the background fluorescence signal. (a) Contact angle on bare standard microscope slide; (b and c) contact angles on standard microscope slide after spin coating with F/ZnO at 2 mg mL^−1^ and 4.6 mg mL^−1^ respectively; (d) background fluorescence on bare standard microscope slide; (e and f) background fluorescence on standard microscope slide after spin coating with F/ZnO at 2 mg mL^−1^ and 4.6 mg mL^−1^ respectively; (g) contact angle on bare amino-slide; (h and i) contact angles on amino-slide after spin coating with F/ZnO at 2 mg mL^−1^ and 4.6 mg mL^−1^ respectively; (j) background fluorescence on bare amino-slide; (k and l) background fluorescence on amino-slide after spin coating with F/ZnO at 2 mg mL^−1^ and 4.6 mg mL^−1^ respectively.

All these measurements were performed before and after the coating. Fig. 2(a–l) shows the typical images recorded for the measurement of the contact angle and for the fluorescence evaluation, where the bare slide refers to the slide before coating. The SEM/EDX results are presented in Fig. S3. The film of F/ZnO QDs increases the surface hydrophobicity in both types of slides, as shown by the images in Fig. 2(a–c) and (g–i), which report the measurements performed before and after coating, respectively. Moreover, in case of the coated slides, the results demonstrate that the amino slide provides a wider contact angle compared to the standard slide (see Fig. 2(h and i)), indicating a poorer nanoparticle deposition in the latter case.

This observation is confirmed by the fluorescence images, where the background signal decreased drastically after coating in case of the amino slide (see Fig. 2(k and l)). This suggests that the F/ZnO layer effectively saturates the slide surface, providing a relatively homogeneous and well-balanced coating distribution. Conversely, in case of the standard microscope slides the F/ZnO layer increased the background fluorescence signal (see Fig. 2(e, f) and S4), which is undesirable for FRET-based applications. These behaviors have been consistently observed at both tested F/ZnO QDs concentrations, 2 mg mL^−1^ and 4.6 mg mL^−1^. The SEM/EDX analysis confirmed the uniformity of the F/ZnO film on the glass slides as illustrated in Fig. S3. In particular, the coatings obtained on the amino slides with the 2 mg per mL solution exhibited a higher zinc concentration. Therefore, considering the lower fluorescence background and the higher concentration and homogeneity of the QDs, we selected the amino slides for the 2D FRET nanoplatform.

**Fig. 3:**
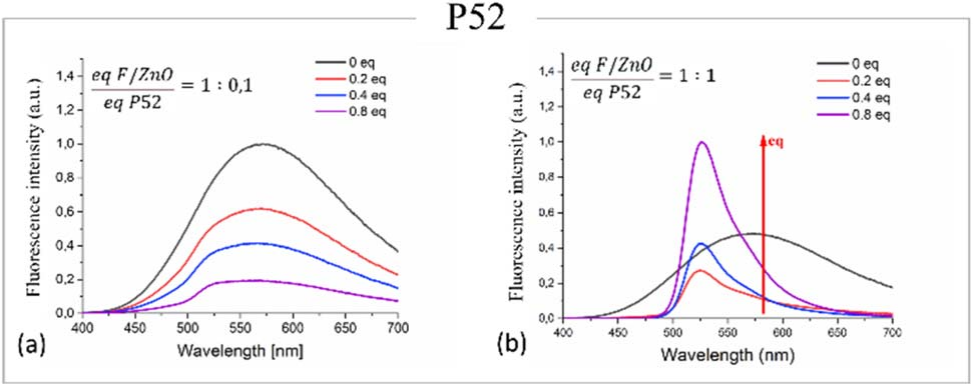
(a) Normalized emission spectra of F/ZnO QDs at different equivalents of P52 with a 1 : 0.1 ratio; (b) normalized emission spectra of F/ZnO QDs at different equivalents of P52 with a 1 : 1 ratio.

We investigated the affinity of the labelled peptides P52 and P11 with the F/ZnO, by evaluating the emission properties of the F/ZnO in solution added with different concentrations of each peptide. Since the maximum adsorption wavelength was exhibited at 350 nm (see Fig. 1(c)), this was used to excite the solution and to evaluate the fluorescence signal generated by the interaction between F/ZnO and each peptide, and Fig. 3 presents the corresponding results in case of the peptide P52.

Fig. 3(a) shows that at the equivalent ratio F/ZnO : P52 = 1 : 0.1 the profile of the emission signal was relatively quenched and presented non-significant variations even at increasing amounts of peptide equivalents. On the contrary, at the equivalent ratio F/ZnO : P52 = 1 : 1 a clear FRET phenomenon was detected since the fluorescence of the QDs was quenched and the peptide signal at 525 nm increased, indicating a specific interaction between F/ZnO and P52, as shown in Fig. 3(b). The results concerning the negative control represented by the labelled peptide P11 are shown in Fig. S5. In case of the ratio F/ZnO : P11 = 1 : 0.1 (see Fig. S5(a)) there was just a dilution of the emission signal of the F/ZnO and no significant emission peak from the peptide was detected.

**Fig. 4:**
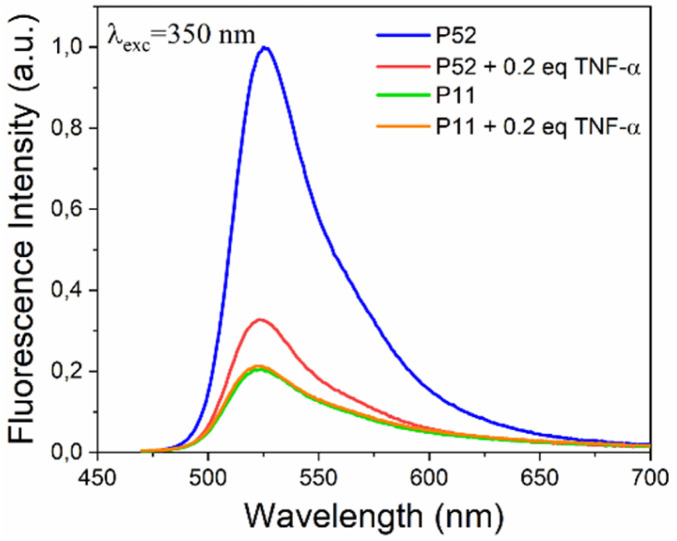
Normalized emission spectra of FITC-labelled P52 and P11 peptides (QDs : peptide = 1 : 1) with and without 0.2 equivalents of TNF-α, showing fluorescence changes upon specific interaction.

Conversely, Fig. S5(b) reported the result for the ratio F/ZnO : P11 = 1 : 1, where the FRET mechanism is observed slightly. The signal of the F/ZnO was indeed quenched only from equivalents higher than 0.2, indicating as the F/ZnO QDs possess a higher interaction with the P52 peptide. Our hypothesis is that the P11 peptide tends to aggregate, undergoing conformational changes that prevent it from interacting with F/ZnO, specially making its binding site less accessible.^51^ As a result, the aggregated P11 is unable to properly associate with the F/ZnO QDs surface due to steric hindrance or loss of the necessary binding conformation hindering the planned interaction. In addition, based on these results, we also investigated the behaviour of the fluorescence response for each labelled peptide P52 and P11 in bare solution and with the addition of 0.2 equivalents of the target protein TNF-α, to evaluate the specificity of the peptide analyte interaction in solution. Fig. 4 shows the resulting spectra. In particular, the emission spectrum of FITC-labelled P52 (dark blue curve) showed a strong peak at ∼525 nm, which significantly decreased upon the addition of the 0.2 equivalents of TNF-α (light blue curve). This quenching was consistent with energy transfer due to complex formation, confirming a specific binding between P52 and TNF-α. In contrast, the control peptide P11 (red curve) exhibited only a low emission signal, and the addition of TNF-α (orange curve) resulted in minimal change, indicating poor or non-specific binding. These results validated the selective affinity of P52 for TNF-α and demonstrated the negligible interaction with P11. It is noteworthy that this kind of investigation was not possible at lower concentrations of TNF-α.

This is due to the limitations typical of the solution environment, where aggregation and precipitation effects become significant at lower concentrations, thus compromising both the spectral resolution and the assay reproducibility. This highlights the necessity of using a surface-confined strategy, such as the 2D F/ZnO p-jet nanoplatform proposed here for a stable and reliable FRET detection at low analyte concentrations.

### Detection of TNF-α by the 2D-FRET nanoplatform

**Fig. 5:**
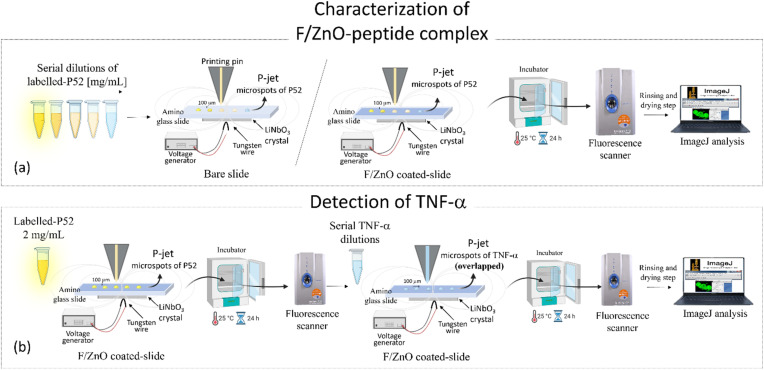
Schematic view of the general workflow for the detection of TNF-α through our 2D-FRET nanoplatform. (a) Printing of ten replica of peptide microspots at a concentration of 2 mg mL^−1^, 0.8 mg mL^−1^, 0.6 mg mL^−1^, 0.4 mg mL^−1^, 0.2 mg mL^−1^ by the p-jet technique on the amino glass slide (bare as control or F/ZnO coated) and next step of incubation, rinsing and drying of the printed slide ready for scanner acquisition and successive analysis of the fluorescence signal produced by the 2D confined peptide-F/ZnO complex; (b) complete 2D nanoplatform for TNF-α detection.

**Fig. 6:**
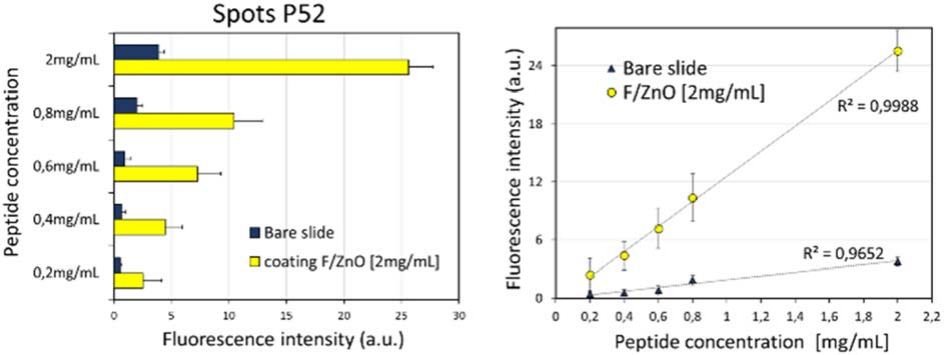
(Left) mean fluorescence values of p-jet micro-spots in the case of 2 mg mL^−1^, 0.8 mg mL^−1^, 0.6 mg mL^−1^, 0.4 mg mL^−1^, and 0.2 mg mL^−1^ of P52, both bare slide (dark blue columns) and the functionalized slide with F/ZnO (yellow columns), obtained *via* a fluorescence scanner. (Right) linear dependence of peptide concentration with fluorescence signal with an *R*^2^ = 0.99 for the F/ZnO-coated slide and *R*^2^ = 0.96 for the bare slide. The SNR was >3 for all the spots.

To confirm the correct interaction of the p52 peptide with the solid-supported ZnO QDs, FTIR spectra were recorded and are shown in SI (Fig. S6). The p52-functionalized F/ZnO films display clear amide I (∼1650 cm^−1^) and amide II (∼1540 cm^−1^) bands, absent in bare glass or F/ZnO-only controls, indicating successful peptide binding. Shifts and slight broadenings of these bands relative to free peptide signatures, along with changes in the O–H/N–H stretching region (3000–3600 cm^−1^) and subtle modifications in the Zn–O vibration region (450–550 cm^−1^), are consistent with coordination of peptide donor groups (*e.g.* –NH_2_, –COO^−^) to surface Zn^2+^ sites. These observations provide qualitative evidence of stable peptide immobilization on ZnO a key requirement for achieving a controlled donor/acceptor arrangement in the solid-supported FRET platform. Fig. 5 shows the schematic view of the general workflow adopted here for the characterization of our 2D-FRET nanoplatform and for the application for detecting the biomarker TNF-α. First, we tested the performance of this innovative nanoplatform by investigating its ability to support effective fluorescence emission. To this aim we prepared serial dilutions of the labelled P52 at the following concentrations in Milli-Q water: 2 mg mL^−1^, 0.8 mg mL^−1^, 0.6 mg mL^−1^, 0.4 mg mL^−1^, 0.2 mg mL^−1^. Following the scheme in Fig. 5(a) we produced ten replicates of microspots for each dilution on two different glass slides, (i) on the amino slide coated with a layer of F/ZnO at 2 mg mL^−1^ and (ii) on the bare amino slide without coating, as control. This was achieved by using the p-jet technique described in Material and methods section,^27,30,42,52,53^ which enabled the formation of well localized and confined microspots of the peptide onto the film of F/ZnO and on the control surface. After 24 h incubation at 25 °C, the slide with printed microspots was subjected to standard rinsing and drying to remove the excess of unbound reagents. Finally, the slide was inserted into the scanner to record the fluorescence images of the microspots that were then analysed by ImageJ, with the aim at evaluating the signal generated by the 2D confined peptide-F/ZnO complex. Fig. 6 shows the resulting data. Fig. 6(a) presents the behaviour of the mean fluorescence intensity for each concentration of labelled P52 retrieved as a mean value over the ten replicated spots subtracted by the blank signal (microspots with only buffer). It is clearly visible the concentration-dependent behaviour of the intensity in both kinds of slides, coated (yellow bars) and un-coated (blue bars), with the higher signal detected for P52 at 2 mg mL^−1^, thus demonstrating the reliability of the technique. The fluorescence intensity of the peptide was significantly higher in case of the F/ZnO coated slide compared to the bare slide, confirming the role of the F/ZnO layer in enhancing the emission signal through the FRET effect.

**Fig. 7:**
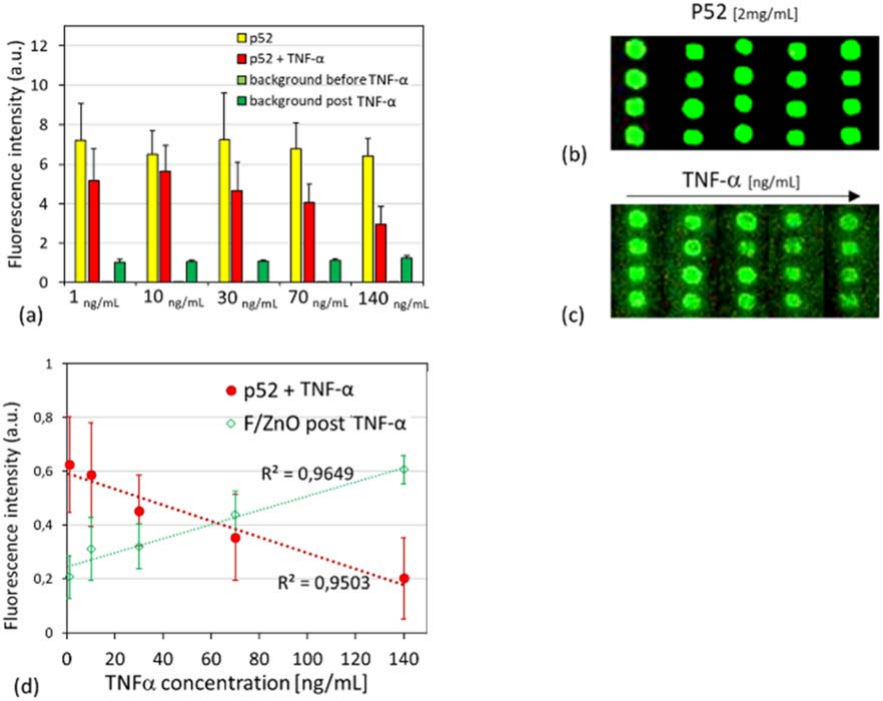
(a) Comparison histogram of the fluorescence intensity of the spots before (in red) and after (in yellow) the overlap of TNF-α spots, and of the background before (in light green) and after (in dark green) the overlap of the analyte. (b) and (c) scanner images showing the homogeneity and repeatability of P52 spots at the fixed concentration of 2 mg mL^−1^ and overlapping TNF-α spots at different concentrations, each at a single column; (d) mean fluorescent intensity of p-jet spots of TNF-α with a linear trend in the range of 1–140 ng mL^−1^ (red points); mean fluorescence intensity of the background around P52 spot in presence of the analyte (green points); Table 1. Limit of Detection (LoD) and Limit of Quantification (LoQ) of the TNF-α calculated from the linear regression of the normalized data [ng mL^−1^].

In fact, the p-jet technique allowed us to assess the P52 immobilization as well as the subsequent increase in the fluorescence signal brought on by the FRET interaction with the F/ZnO nanoparticles on the glass surface. A linear correlation between the fluorescence intensity and the peptide concentration was observed on both surfaces, with an excellent coefficient of determination (*R*^2^ = 0.99) for the F/ZnO-coated slide and a slightly lower value (*R*^2^ = 0.96) for the bare slide. These results confirmed the reliable fluorescence enhancement provided by the F/ZnO layer and its suitability for quantitative detection in our innovative surface-confined 2D nanoplatform. Indeed, the fixed spatial arrangement determined by the F/ZnO film reduced drastically the random diffusion effects typically occurring in a solution-based reaction environment. The proximity of donor and acceptor molecules in our 2D-FRET nanoplatform confines significantly the FRET signal, resulting in highly reproducible measurements with reduced background noise.^9^ Once assessed the reliability and stability of the 2D confined F/ZnO–peptide complex, we evaluated the possibility to use our innovative nanoplatform for quantitative biomarker detection. To this aim we prepared a stock solution of TNF-α in PBS 1× pH 7.4 at a concentration of 0.14 mg mL^−1^ and then 5 serial dilutions at 140 ng mL^−1^, 70 ng mL^−1^, 30 ng mL^−1^, 10 ng mL^−1^, and 1 ng mL^−1^. Following the scheme in Fig. 5(b) we first printed on the F/ZnO coated amino slide 50 replica of p-jet microspots of the labelled P52 at 2 mg mL^−1^, *i.e.* the concentration that produced the highest signal intensity in the previous characterization procedure. After 24 h incubation at 25 °C we recorded the fluorescence image as control. Successively, we mounted the slide again in the p-jet setup and we printed ten replicas of each dilution of TNF-α on the pre-existing P52 microspots. Another incubation step overnight was performed to ensure the binding between the immobilized F/ZnO–peptide complex and the target protein TNF-α. Then, we rinsed the slide with Milli-Q water and dried by nitrogen gas flow to remove unbound molecules and to minimize the background noise. Finally, we recorded the fluorescence images by the scanner, and we used ImageJ for the analysis. All the results are shown in Fig. 7. In particular, Fig. 7(a) compares the fluorescence intensity registered before and after TNF-α overlapping. The yellow columns show that the signal is almost constant, coherently with the constant concentration used for P52 spots generation, while the red columns show a clear decreasing trend with the increasing analyte's concentration. Moreover, the background signal around the spots clearly increases in presence of the target TNF-α because it tends to increase the distance between the peptide and the F/ZnO QDs with a consequent increase of the native fluorescence of the QDs and decrease of the FRET emission. These observations are well confirmed by Fig. 7(b and c), which shows the typical scanner images recorded after printing the P52 microspots and after overlapping the microspots of TNF-α at serial dilutions, respectively. We reported the results of the quantitative analysis in Fig. 7(d), where the red points correspond to the microspots with TNF-α and the green ones to the background around the spots in presence of the analyte. A decrease in P52 fluorescence intensity by an average of 73% was observed in presence of the analyte, linearly proportional to its increasing concentration, accompanied by a corresponding increase of approximately 98% in the fluorescence signal of the surrounding F/ZnO QDs due to the above-mentioned FRET attenuation.

**Fig. 8:**
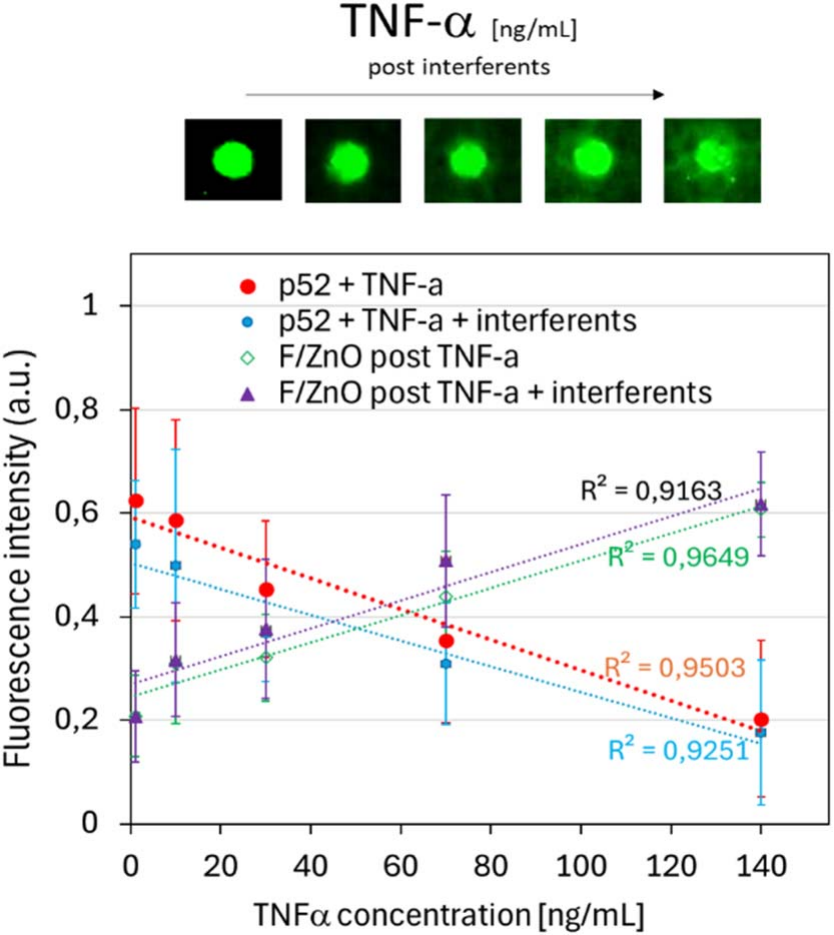
(Top) Scanner images of P52 microspots after overlapping TNF-α spots at increasing concentrations (1–140 ng mL^−1^) in the presence of interferents (BSA 100 ng mL^−1^ and p-Tau181 2 pg mL; final concentrations), prepared in artificial human urine (Biochemazone, BZ325) diluted 1 : 10 (v/v) in 1× PBS. (Bottom) Mean fluorescence intensity of P52 p-jet microspots as a function of TNF-α concentration in the absence (red points) and presence (blue points) of interferents; mean fluorescence intensity of the background around the spots after analyte exposure in the absence (green points) and presence (purple points) of interferents. Dotted lines indicate linear regression fits (*R*^2^ values reported). Table 2. Limit of Detection (LoD) and Limit of Quantification (LoQ) calculated from the linear regression [ng mL^−1^].

The LoD and LoQ value are also reported, in Table 1, showing values of (31 ± 4) ng mL^−1^ and (94 ± 12) ng mL^−1^, respectively. These findings demonstrate that FRET efficiency was greatly enhanced by the localized co-accumulation of both probe and analyte within the p-jet-generated microspots. Additionally, the fixed spatial arrangement of donor and acceptor molecules minimized random scattering and diffusion, major limitations of solution-based assays, resulting in highly repeatable and sensitive fluorescence signals. In line with point-of-care requirements, we further assessed assay robustness and selectivity under more challenging conditions by repeating the TNF-α calibration in a surrogate urine matrix and in the presence of representative protein interferents; under these conditions, the calibration trend was preserved and the LoD/LoQ remained comparable (variation <10%) to the interference-free assay (see Fig. 8 and Table 2). As a negative control, the same test was performed using the peptide P11 at the highest concentration tested of 140 ng mL^−1^ (see Fig. S7(a)). The results consistently demonstrated a lack of affinity between P11 and TNF-α, even at elevated concentrations, with a complete loss of fluorescence signal attributable to the rinsing step with Milli-Q water, suggesting that no FRET mechanism had occurred. To further confirm the specificity and correct functioning of the FRET system, the effect of the PBS buffer (used as analyte vehicle) on the fluorescence intensity of both the peptide and nanoparticle layer was also evaluated (see Fig. S7(a)). The measurements revealed a minor decrease in P52 fluorescence (∼10%) after rinsing, an unremarkable result attributed to the removal of loosely bound material rather than any specific interaction. The same protocol was applied to assess the impact of potential interferents by constructing calibration curves for TNF-α (1–140 ng mL^−1^) in the presence of 100 ng per mL BSA and 2 pg per mL p-Tau181 (final concentrations). To better approximate a complex biological environment, the assay was carried out in artificial human urine (Biochemazone, BZ325) diluted 1 : 10 (v/v) in 1× PBS, following the same acquisition and analysis workflow used for Fig. 7, *i.e.*, quantifying fluorescence on the P52 microspots and in the surrounding F/ZnO background. The results shown in Fig. 8 consistently demonstrated that the surface-confined FRET response was preserved under interference and complex-matrix conditions. In artificial urine (1 : 10 in 1× PBS) supplemented with 100 ng mL^−1^ BSA and 2 pg per mL p-Tau181, P52 spot fluorescence decreased progressively with increasing TNF-α concentration, while the fluorescence signal measured in the surrounding F/ZnO background increased, maintaining the expected complementary behaviour. The corresponding calibration curves retained good linearity (*R*^2^ values reported in the figure), indicating that non-target proteins and matrix components only minimally affected the quantitative readout and supporting the robustness and selectivity of the assay. Without interferents (Table 1), the assay yielded a LoD of (31 ± 4) ng mL^−1^ and a LoQ of (94 ± 12) ng mL^−1^. Under interference conditions (Table 2), both LoD and LoQ remained comparable, showing only a limited change relative to the interference-free case, confirming that matrix components and non-target proteins have a minor impact on analytical sensitivity. These data support the robustness of the proposed surface-confined FRET strategy under interference and complex-matrix conditions. As a precise and repeatable micro-spotting strategy, the p-jet technique offered a promising route for integration into scalable biosensing platforms, enabling reliable fluorescence-based readouts suitable for future diagnostic applications.

**Table 1:** Quantification of LoD and LoQ

Limit of detection (LoD)	Limit of quantification (LoD)
(31 ± 4) ng mL^−1^	(94 ± 12) ng mL^−1^

**Table 2:** Quantification of LoD and LoQ

Limit of detection (LoD)	Limit of quantification (LoD)
(31 ± 4) ng mL^−1^	(94 ± 12) ng mL^−1^

## Conclusions

The ability to detect protein biomarkers at low concentrations plays a key role in understanding disease-related biological responses and guiding therapeutic strategies Traditional FRET assays in solution suffer from uncontrolled donor–acceptor diffusion, fluorophore quenching, and poor reproducibility, which limit their sensitivity and practical utility. To overcome these barriers, we propose here a two-dimensional, solid-supported FRET biosensing nanoplatform that combines a high-precision pyro-electrohydrodynamic jetting (p-jet) technique with fluoride-doped ZnO quantum dots. By exploiting the pyroelectric effect of a LiNbO_3_ crystal, our p-jet method deposited microspots of the affinity peptide P52 and the target analyte TNF-α onto amino-functionalized glass slides uniformly coated with F/ZnO QDs. By using this method, microscale p-jet spots with fixed donor–acceptor spacing and improved photostability were produced. Spectroscopic characterization confirmed optimal spectral overlap between P52 emission and F/ZnO absorption, supporting efficient energy transfer. On F/ZnO–coated slides, P52 fluorescence was up to 10× greater than on bare glass, with a linear concentration response (*R*^2^ = 0.99), demonstrating robust peptide immobilization and quantitative readout. With TNF-α co-spotted, we were able to detect TNF-α in the ng mL^−1^ range, with high specificity by achieving a clear concentration-dependent decrease in peptide fluorescence (up to 73%) and a complementary increase in F/ZnO emission (up to 98%). The nanoplatform reached a limit of detection (LOD) of ≈31 ng mL^−1^, without the need for complex amplification protocols. The platform exhibits a signal-to-noise ratio ≫5, minimal reagent consumption, and room-temperature operation highlighted its reproducibility and scalability. The designed 2D FRET nanoplatform through p-jet on a film of F/ZnO QDs enabled quantitative detection of low-abundance targets, enhanced FRET efficiency, and precise micro spotting, effectively addressing key limitations of conventional solution-based assays. We acknowledge that, without signal amplification, pg mL^−1^ detection is not achievable with the current FRET platform. Nevertheless, the present work establishes a reproducible, and scalable solid-supported FRET architecture, which can be further optimized in future studies toward improved sensitivity. To indicate realistic pathways toward improved analytical performance, we outline possible strategies for future optimization, including the use of brighter fluorophores or higher-quantum-yield quantum dots, integration of nanomaterial-based signal amplification, and sample pre-concentration *via* microfluidic interfaces. This represents, to our knowledge, the first reliable implementation of peptide-based FRET for TNF-α detection on a nanostructured 2D surface. To better approximate a complex biological environment relevant to point-of-care translation, we additionally evaluated the assay in a surrogate urine matrix and assessed specificity in the presence of representative non-target proteins (BSA and p-Tau181). The overall sensor response was on average lower than in interference-free conditions, consistent with partial damping effects from additional matrix proteins; however, the detectability parameters were only slightly affected (LoD ≈ 39 ng mL^−1^), supporting the robustness of the surface-confined FRET architecture under interference and complex-matrix conditions. We believe that our findings would contribute to the advancement of nanostructure-assisted biosensors and would provide a foundation for their integration into portable, point-of-care diagnostic devices capable of targeting a wide spectrum of biomarkers in both clinical and environmental settings.

## Author contributions

Stefania Carbone: investigation, data curation, writing – original draft and formal analysis; Simone Russo: investigation, data curation, writing – original draft and formal analysis; Anna Palma: data curation, analysis, and manuscript revision; Giuseppe Junior Mosca: data curation, analysis, and manuscript revision; Sara La Manna: data curation, analysis, and manuscript revision; Alessia Cugudda: data curation, analysis, and manuscript revision; Sara Coppola: data curation, analysis, and manuscript revision; Pier Luca Maffettone: supervision, funding acquisition, and project administration; Simonetta Grilli: supervision and writing – review and editing; Giuseppe Vitiello: conceptualization, supervision, writing – original draft, and writing – review and editing; and Concetta Di Natale: conceptualization, supervision, formal analysis, investigation, data curation, writing – original draft, and writing – review and editing.

## Conflicts of interest

There are no conflicts to declare.

## Supplementary Material

RA-016-D6RA00144K-s001

## Data Availability

The data that support the findings of this study are available from the corresponding authors upon request. Supplementary information (SI) is available. See DOI: https://doi.org/10.1039/d6ra00144k.
