# Supplementary material for: Surface-confined FRET nanoplatform printed via pyro-EHD jet for stable and reproducible TNF-α detection
Source: RSC Adv. 2026 Apr 20;16(22):20304–15. doi: 10.1039/d6ra00144k (PMC13093259; doi:10.1039/d6ra00144k)
Supplement: RA-016-D6RA00144K-s001 [file RA-016-D6RA00144K-s001.pdf]

Supplementary Information (SI) for RSC Advances.
This journal is © The Royal Society of Chemistry 2026

Surface-Confined FRET Nanoplatform Printed via Pyro-EHD Jet for Stable and Reproducible TNF-α Detection

Stefania Carbone [a,b,†], Simone Russo[a,c,†], Anna Palma[b], Giuseppe Junior Mosca[a], Sara La Manna[d], Alessia Cugudda[d], Sara Coppola [b], Pier Luca Maffettone[a,b], Simonetta Grilli[b,*], Giuseppe Vitiello[a,c*], Concetta Di Natale[a,b,*]

[a] Department of Chemical, Materials and Industrial Production Engineering, University of Naples Federico II, Piazzale Tecchio 80, 80125 Naples, Italy
[b] Institute of Applied Sciences and Intelligent Systems (ISASI), National Research Council of Italy (CNR), Pozzuoli, NA 80078, Italy.
[c] Center for Colloid and Surface Science (CSGI), via della Lastruccia, Sesto Fiorentino, FI 80078, Italy
[d] Department of Pharmacy, University of Naples Federico II, 80131 Naples, Italy

[†] These authors equally contributed to this work.

[*]corresponding authors: giuseppe.vitiello@unina.it concetta.dinatale@unina.it, simonetta.grilli@cnr.it

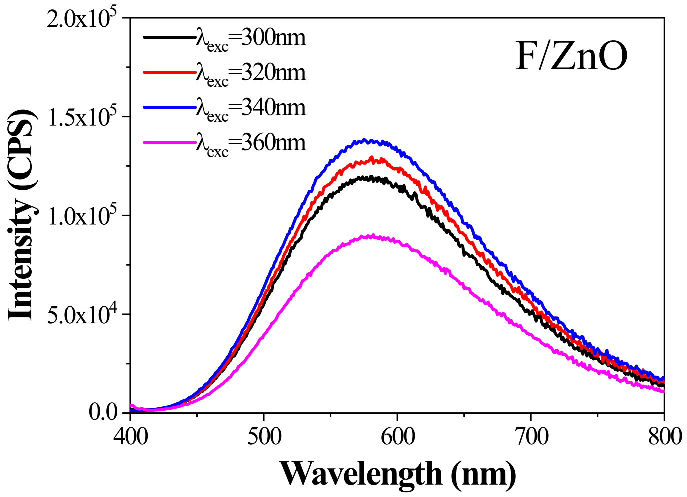

Figure S1: *Emission map of F/ZnO QDs*

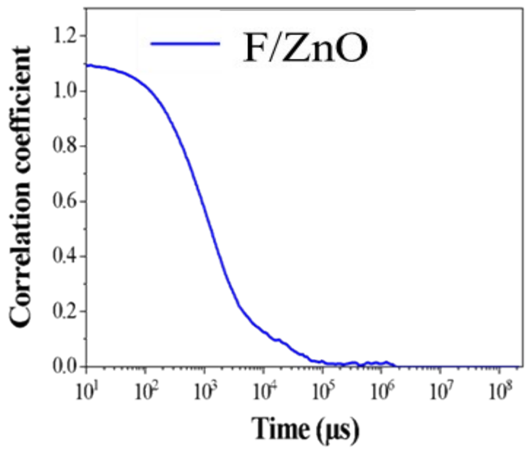

Figure S2: *Result of the DLS measurements on F/ZnO in solution with the behaviour of the autocorrelation function.*

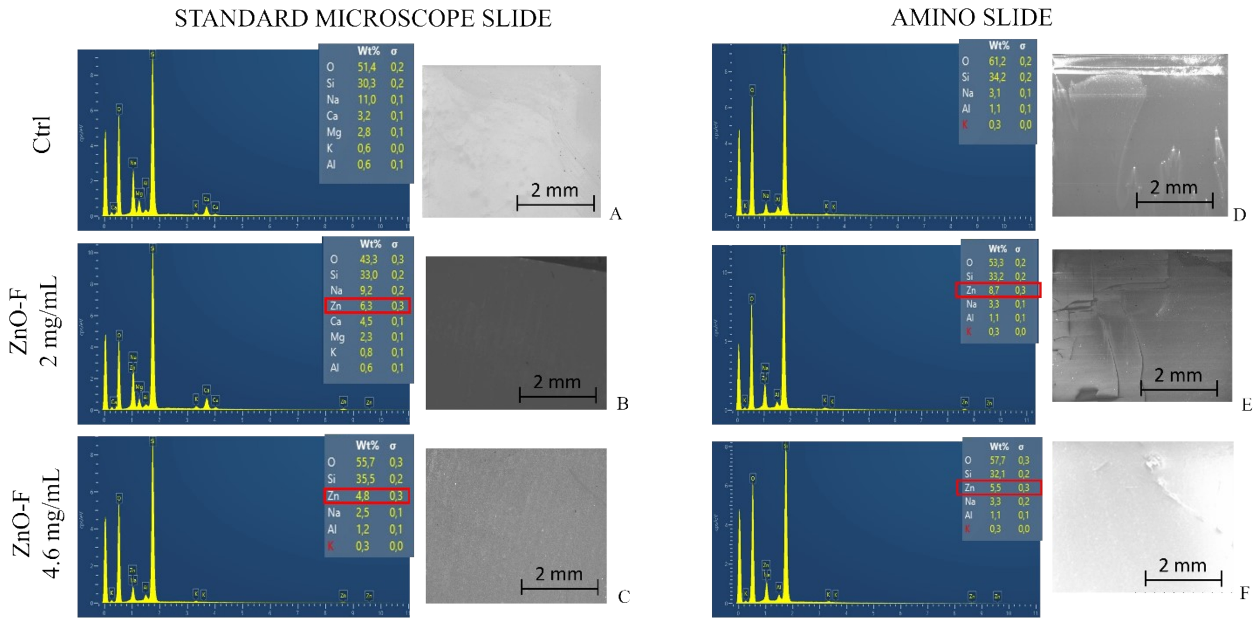

***Figure S3:** SEM and EDX of the respective slides: (A) control microscope standard slide, (B) microscope standard slide 2 mg/mL of F/ZnO, (C) microscope standard slide 4.6 mg/mL of F/ZnO, (D) control amino slide, (E) amino slide 2 mg/mL of F/ZnO, (F) amino slide 4.6 mg/mL of F/ZnO.*

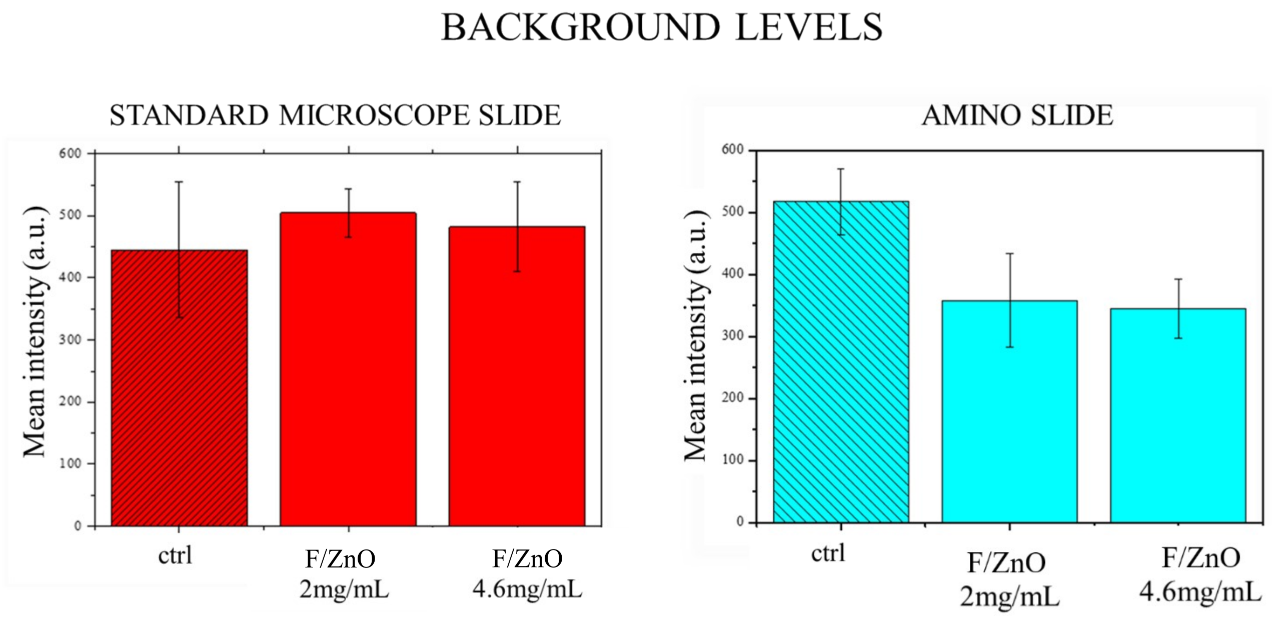

Figure S4: *Background emission intensity of 2D slides*

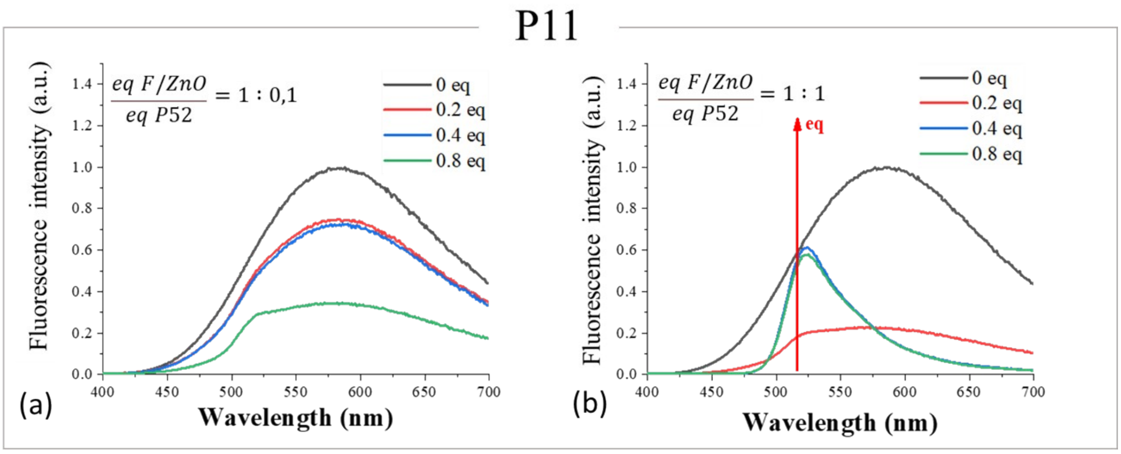

Figure S5: *Normalized mission spectra of F/ZnO QDs at different equivalents of P11 with a 1:0.1 ratio* ***(a)****, normalized emission spectra of F/ZnO QDs at different equivalents of P11 with a 1:1 ratio* ***(b)****.*

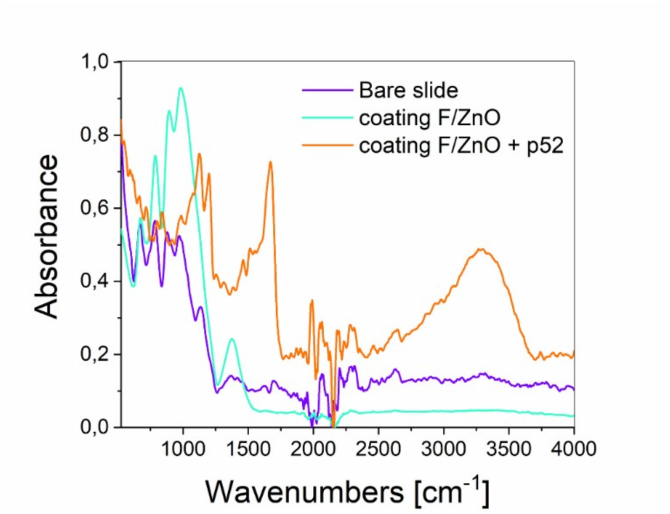

Figure S6. *FTIR spectra of bare glass slide (purple), F/ZnO-coated slide (cyan), and F/ZnO + p52 functionalized slide (orange). The spectrum of the bare slide serves as background reference. The F/ZnO-coated slide shows minimal absorption features in the amide region, while the F/ZnO + p52 sample displays pronounced amide I (~1650 cm⁻¹) and amide II (~1540 cm⁻¹) bands, indicating successful immobilization of the p52 peptide on the ZnO surface. Compared to typical free-peptide signatures, these bands are slightly broadened and shifted, consistent with perturbation of C=O and N–H moieties due to coordination with Zn^{2+} surface sites. Additionally, the O–H / N–H stretching region (3000–3600 cm⁻¹) becomes more intense and structured upon peptide binding, suggesting altered hydrogen-bonding and possible partial coordination of amine groups. Subtle changes in the Zn–O vibration region (450–550 cm⁻¹) further support interaction between peptide donor groups and surface Zn^{2+}. Collectively, these spectral features constitute qualitative evidence of peptide adsorption and coordination-based binding on the F/ZnO surface, an essential basis for the controlled donor–acceptor spacing enabling the solid-supported FRET configuration.*

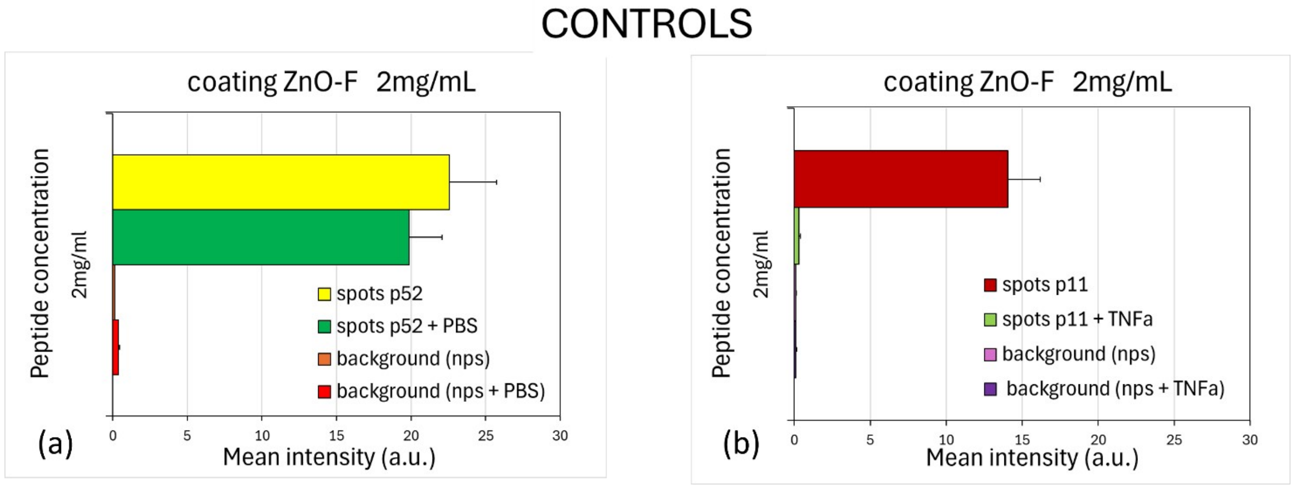

Figure S7: (a) *Mean fluorescence values of p-jet micro-spots in the case of the concentrations of P52 (2 mg/mL) and background levels of the F/ZnO coated amino slide, after spotting and after the rinsing of the slide with PBS, used as a vehicle for the analyte.* ***(b)*** *Mean fluorescence values of p-jet micro-spots in the case of the concentrations of P11 (2 mg/mL) and background levels of the F/ZnO coated amino slide, after spotting 140 ng/mL of TNF-α on P11 and after the rinsing of the slide with PBS. The fluorescence data were obtained as mean values over ten replicates of spots and subtracted by the control (blank spots). The SNR was >3 for all the spots.*
